# Supplementary material for: A CDK1 phosphorylation site on Drosophila PAR-3 regulates neuroblast polarisation and sensory organ formation
Source: eLife. 2024 Jun 13;13:e97902. doi: 10.7554/eLife.97902 (PMC11216751; doi:10.7554/eLife.97902)
Supplement: Figure 7—source data 1. — (File 7B left panel colour) Full western blot corresponding to Figure 7B. left panel. Dual-color LICOR fluorescent image of 800 nm (green, anti-Baz-pS180 antibody) and 700 nm (red, ladder) channels shown in left panel. (File 7B left panel bw) Same image in black and white. (File 7B right panel colour) Full western blot corresponding to Figure 7B. right panel. Membrane stripped and reprobed with anti-GST antibody. Dual-color LICOR fluorescent image of 800 nm (green, anti-GST antibody) and 700 nm (red, ladder). (File 7B left panel bw) same image in black and white. [file elife-97902-fig7-data1.zip › Figure 7 - source data/Raw blots with labels.pdf]

Supplementary western blot images corresponding to Figure 7B.

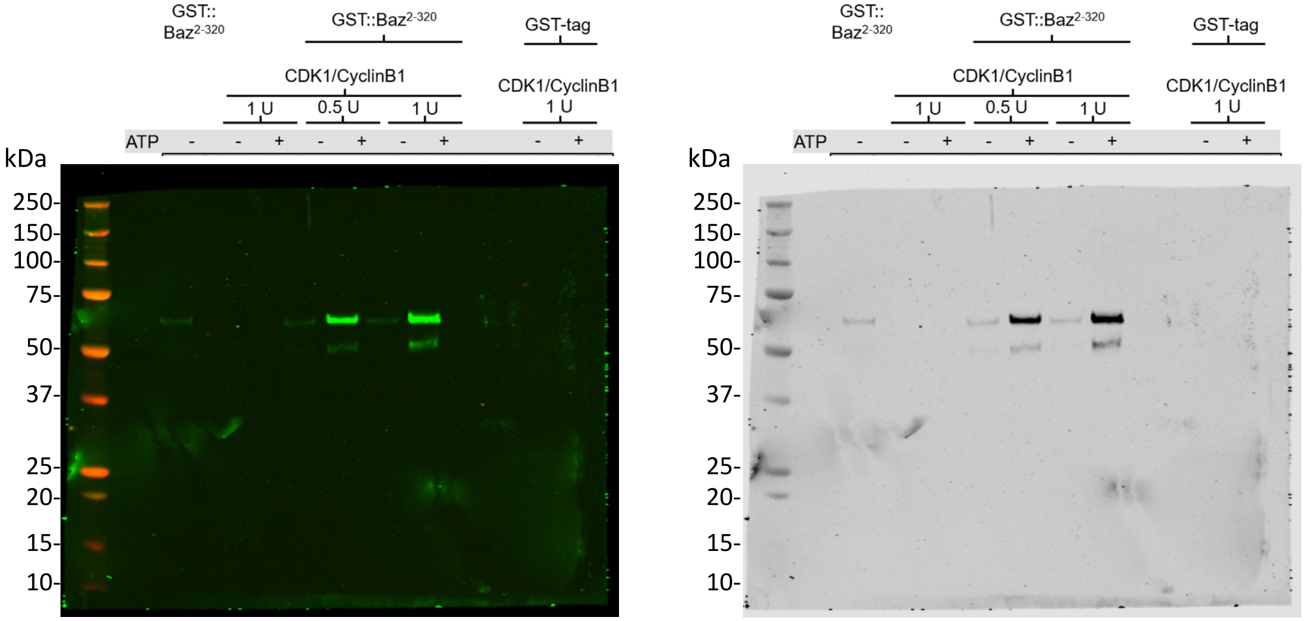

1. Full western blots corresponding to Figure 7B "left panel". Left, dual-colour LICOR fluorescent image of 800nm (green, anti-Baz-pS180 antibody) and 700nm (red, ladder) channels. Right, same image in black and white.

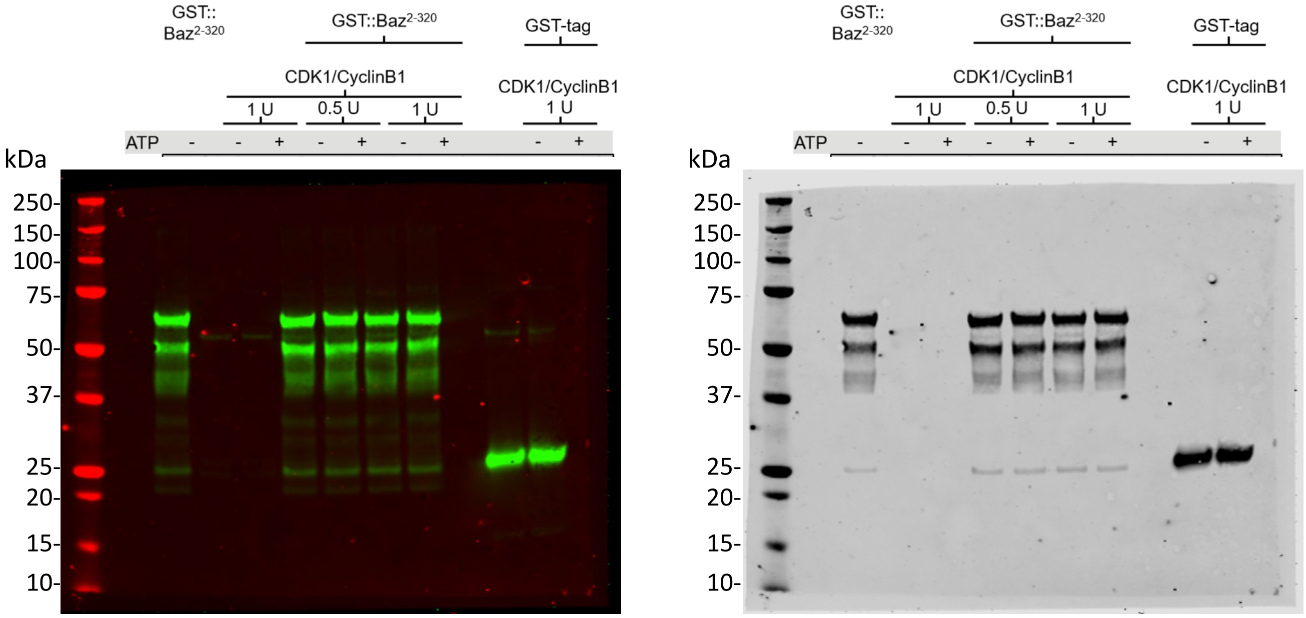

2. Full western blots corresponding to Figure 7B "right panel". Membrane stripped and reprobed with anti-GST antibody. Left, dual-colour LICOR fluorescent image of 800nm (green, anti-GST antibody) and 700nm (red, ladder) channels. Right, same image in black and white.
